# Converging rapid deployment prostheses with minimal access surgery: analysis of early outcomes

**DOI:** 10.1186/s13019-021-01739-w

**Published:** 2021-12-27

**Authors:** M. Yousuf Salmasi, Kristo Papa, David Mozalbat, Muhammad Ashraf, Alicja Zientara, Ishaan Chauhan, Nikoleta Karadatkou, Thanos Athanasiou, Isabelle Roussin, Cesare Quarto, George Asimakopoulos

**Affiliations:** 1 Department of Surgery, Imperial College London, 10th Floor QEQM, Praed Street, London, W2 1NY UK; 2 Royal Brompton and Harefield Foundation Trust, London, UK; 3 East and North Hertfordshire NHS Trust, Stevenage, UK

**Keywords:** Mini-sternotomy, Surtureless, Perceval, Aortic valve replacement

## Abstract

**Background:**

Sutureless prostheses may have added benefit when combined with minimal access surgery, although this has not been fully assessed in the literature. This study aims to provide a comparative analysis of the Perceval valve comparing median sternotomy (MS) with mini-sternotomy (MIS).

**Methods:**

A retrospective analysis of prospectively collected data was conducted for all isolated aortic valve replacement (AVR), using the Perceval valve, for severe aortic stenosis cases in the period 2014 to 2019. Patients undergoing concomitant valve or revascularisation surgery were excluded.

**Results:**

A total of 78 patients were included: MS group 41; MIS group 37. Operatively, bypass times were comparable between MS and MIS groups (mean 89.3 vs 83.4, *p* = 0.307), as were aortic cross clamp times (58.4 vs 55.9, *p* = 0.434). There were no operative deaths or new onset post-operative neurology. MIS was a predictor of reduced stay in the intensive care unit (coef − 3.25, 95% CI [− 4.93, − 0.59], *p* = 0.036) and hospital stay overall (*p* = 0.004). Blood transfusion units were comparable as were the incidence of heart block (n = 5 vs n = 3, *p* = 0.429) and new onset atrial fibrillation (n = 15 vs n = 9, *p* = 0.250). Follow-up echocardiography found a significant improvement in effective orifice area, left ventricular dimension and volume indices, and LVEF (*p* > 0.05) for all patients. Multivariate analysis found mini-sternotomy to be a predictor for reduced LV diastolic volume (coef − 0.35, 95% CI [− 1.02, − 0.05], *p* = 0.05).

**Conclusions:**

The combination of minimal access surgery and sutureless AVR may enhance patient recovery and provide early LV remodelling.

## Introduction

The risk of complications from conventional sternotomy for aortic valve replacement (AVR) centre around deep sternal wound infection, sternal dehiscence and pain [1]. Comparative studies have found that upper hemi-sternotomy for AVR (mini-AVR) is associated with reduction in post-operative mediastinitis, intensive care unit stay and time to return to work compared to full sternotomy [2, 3].

Despite this, registries show that the rate of conventional sternotomy for AVR remains far more common compared to minimally invasive approaches [4]. This may reflect two factors: (1) many trials have demonstrated clinical equipoise between the use of conventional sternotomy and mini-access procedures for isolated AVR, including similar rates of survival, functional class echocardiographic outcomes when similar prostheses are used [5, 6]. (2) The increased learning curve associated with a more challenging procedure in a reduced operative field [7]. One proposed method to potentiate mini-AVR is the use of rapid deployment, or sutureless, valve prostheses in combination. The elimination of annular stitches and hand tying in a small surgical field can potentiate the operative process.

In a randomised prospective study (n = 100), Borger and colleagues [8] found that patients undergoing upper hemisternotomy and AVR with the Edwards Intuity valve had reduced cross clamp time and improved haemodynamic function compared with full sternotomy. However, the comparability of the cohorts was limited by the sternotomy group receiving conventional sutured prostheses. There have been no randomised studies comparing the additive effect of mini-AVR and the use of sutureless valves in comparable cohorts using the same prosthesis design.

Given the inability of mini-AVR or sutureless valves to widely penetrate general adult cardiac surgical practice, the aim of this study was to compare the outcomes of AVR for the treatment of aortic stenosis using the Perceval S valve (Sorin Group Italia Srl, Saluggia, Italy), performed via two types of access: (1) conventional sternotomy; or (2) mini-sternotomy.

## Methods

Perioperative data was retrospectively analysed from a prospectively collated database at a single cardiothoracic institution between 2014 and 2020. Due to the retrospective nature of the study, the requirement for ethical approval was waivered by the Research Ethics Office at the Royal Brompton and Harefield Foundation Trust.

Only patients undergoing isolated AVR with a Perceval valve, and no added concomitant procedures, were included. Patients were subclassified as having (1) median sternotomy (MS); or (2) mini-sternotomy (MIS). Data was collated from the practice of two operating surgeons at a single centre.

### Procedure

In the MS group, a standard midline incision from sternal notch to the xiphoid process was performed with central division of the sternum, and midline pericardiotomy. Institution of cardiopulmonary bypass (CPB) was performed via cannulation of the distal ascending aortic and insertion of a dual stage venous cannula in the right atrium. Antegrade cold blood cardioplegia was the commonly used technique, as well as direct ostial infusion.

For operative access in the MIS group, a “J” incision from the sternal notch caudally, curving into the right third or fourth intercostal space. The reduced space necessitated the use of slightly different CPB approaches in order to arrive to the stage that the aortic valve is replaced in a safe and effective method, with the main following differences:

1. LV venting performed through the right superior pulmonary vein, otherwise through the main pulmonary artery, where access to the RSPV is difficult.
2. Administration of retrograde cardioplegia is entirely avoided. Only antegrade or direct routes were used.
3. Venous cannulation of the femoral vein could be used as a bailout option where access to the right atrium was significantly limited.

With the aortic cross clamp applied, the procedure was relatively similar in both groups. A transverse aortic incision was made approximately 3–4 cm above the right coronary ostium. This location was important to allow adequate anchoring of the prosthesis at in the supra-annular region. Following native valve excision and annular decalcification, sizing was performed to match the annular dimension without oversizing.

### Implanting the prosthesis

The suture guide eyelets present on the inflow ring of the Perceval prosthesis allowed for precise placement during implantation. The chosen valve was prepared, placed on the deployment device using a collapsing system designed for the chosen valve size. The valve was reduced in size but not crimped to avoid leaflet tissue damage.

Guiding sutures were placed in a symmetric fashion at the nadirs of each sinus. Then these sutures were passed through three small loops located at the level of the annulus of the valve. The valve was then advanced into the aortic root using the guiding sutures and released. The balloon was inflated within the prothesis (4 atmospheres pressure) while gently irrigating with warm saline for 30 s.

### Closure

The aorta was closed with a semicontinuous double-layered 4-0 monofilament suture. Intraoperative transoesophageal Echocardiogram (TOE) after weaning the patient from cardiopulmonary bypass was routine for all patients to ensure a well-seated prosthesis. A single pericardial drain was used for MIS patients, whilst two drains were the protocol for MS patients.

Patients were managed post-operatively according to a structured protocol in an overnight recovery unit and planned for early extubation within 6 h. All patients received a pre-discharge trans-thoracic echocardiogram as part of institutional practice.

### Data collection

The cardiac surgical database is locally managed and centrally overseen at a national level, following national guidelines for minimal perioperative data collection, including pre-operative co-variates, detailed operative characteristics and post-operative care, including the record of short-term complications. Early (6–12 months) outcomes were assessed based on echocardiographic findings and grading of aortic regurgitation.

### Statistical analysis

Results were analysed and presented as means and standard deviations. Pre-operative covariates were assessed for normal distribution using the Shapiro–Wilk test. Between group characteristics were assessed for statistical differences using the Student T test or Wilcoxon Rank Test for non-parametric variables. Multivariate logistic regression models were constructed to assess the influence of a variety of covariates on short term outcomes. Linear regression was used to assess the influence of covariates on parametric outcomes, namely hospital stay. Adjusted odds ratio with 95% confidence interval (CI) of binary outcomes were calculated. Statistical analyses were conducted using the Stata 13.0 software (Stata Corp., College Station, TX, USA).

## Results

During the study period, 185 patients underwent AVR with the Perceval valve. Of these, 78 cases were isolated AVR: MS group n = 41, MIS group n = 37.

### Pre-operative covariates

Both cohorts were comparable for pre-operative covariates in several aspects (Table 1). Age was similar between both groups (MS vs MIS, mean ± standard deviation: 73.1 ± 10.2 vs. 69.5 ± 8.2, *p* = 0.822). Female patients in MS (19/41, 46%) compared to MIS (18/37, 49%) was also not different (*p* = 0.442). Body surface area in MS (1.98 ± 0.34) was similar to the MIS group (1.92 ± 0.40) (*p* = 0.265).

**Table 1:** Pre-operative characteristics of patients undergoing AVR for AS with the Perceval valve, either via mini-sternotomy or full sternotomy

	Median sternotomy (n = 41)	Mini-sternotomy (n = 37)	*p* value
Age	73.1 ± 10.2	69.5 ± 8.2	0.822
Female gender	19	18	0.442
Body surface area	1.98 ± 0.34	1.92 ± 0.40	0.265
Body mass index	29.4 ± 5.3	29.0 ± 5.3	0.393
Diabetes	10	9	0.996
Hypertension	28	21	0.381
Atrial Fibrillation	5	4	0.935
Hypercholesterolaemia	25	17	0.254
Chronic obstructive airway disease	5	3	0.756
Renal failure	2	2	0.968
Euroscore	1.79 ± 0.92	1.34 ± 0.50	0.432
*Echocardiography*			
Aortic valve area (cm^2^)	0.83 ± 0.43	0.78 ± 0.25	0.750
Peak gradient (mmHg)	77.3 ± 32.2	85.0 ± 23.5	0.194
LVEF (%)	56.3 ± 10.6	60.1 ± 7.2	0.361
LV end diastolic diameter (mm)	42.7 ± 14.7	45.2 ± 5.3	0.741
LV end systolic diameter (ml)	12.8 ± 2.4	12.4 ± 2.2	0.310

Co-morbidities were also similar between cohorts. Diabetes in MS (10/41, 24%) compared to MIS (9/37, 24%), hypertension in MS (28/41, 68%) compared to MIS (21/37, 57%), atrial fibrillation (5/41 in MS vs 4/37 for MIS, *p* = 0.935). The mean Euroscore was similar between groups (1.79 in MS vs 1.34 in MIS, *p* = 0.432).

Severity of aortic stenosis was also comparable, as evidenced by pre-operative echocardiographic data: aortic valve area for MS was 0.83 ± 0.43cm2 and for MIS 0.78 ± 0.25 cm^2^ (*p* = 0.738), peak gradient for MS was 77.3 ± 32.2 mmHg and for MIS 85.0 ± 23.5 mmHg (*p* = 0.194). Left ventricular function and dimensions were also similar between the two cohorts (*p* > 0.05).

### Operative parameters

Operatively (Table 2), bypass times were comparable between MIS and MS groups (mean 89.3 vs 83.4, *p* = 0.307), as were aortic cross clamp times (58.4 vs 55.9, *p* = 0.434). The least frequently used valve size was S (12/78, 15%) followed by XL (13/78). The most frequently used valve size was M (29/78, 37%) followed by L (24/78, 31%). There was no statistical difference between the two cohorts in the valve size used (*p* = 0.703).

**Table 2:** Operative parameters

	Median sternotomy (n = 41)	Mini-sternotomy (n = 37)	*p* value
Valve size			0.703
S	6	6	
M	15	14	
L	13	11	
X3	7	6	
Cardiopulmonary bypass time (min)	89.3 ± 47.2	83.4 ± 25.9	0.307
Cross clamp time (min)	58.4 ± 36.3	55.9 ± 20.5	0.434

### Short-term outcomes

There were no incidences of operative mortality or stroke (Table 3). Amongst both groups, only one patient required haemofiltration (MIS cohort), and two patients suffered from post-operative pneumonia (one from each group). The number of patients requiring transfusion were similar between both cohorts (MS vs MIS: 45% vs 31%, *p* = 0.195) as was the number of units transfused: (median, interquartile range), MS 1 (0–3), MIS 1 (0–3) (*p* = 0.331). The length of stay in the intensive care unit (ICU) was also similar: MS vs MIS in days, 3 (2–4) versus 3 (2–6) (*p* = 0.240).

**Table 3:** Short term outcomes

Outcome	Median sternotomy (n = 41)	Mini-sternotomy (n = 37)	*p* value
Resternotomy	2	1	0.622
Pneumonia	1	1	NS
New onset AF	15	9	0.250
Heart block	5	3	0.429
Patients requiring transfusion	18	13	0.310
Units transfused	1 (0–3)	1 (0–3)	0.331
Haemofiltration	0	1	NS
Mortality	0	0	NS
Stroke	0	0	NS
ICU stay (days)	3 (2–4)	3 (2–6)	0.358
Hospital stay	9 (7–13)	7 (6–8)	0.009

*p*-values result from non-parametric nests

The incidence of new onset atrial fibrillation in the MS group was higher than the MIS group (42% vs 24%) although this did not reach significance (*p* = 0.250). The incidence of heart block was also higher in the MS group compared to MIS (12% vs 8%) although this difference was also non-significant (*p* = 0.429).

Using regression analysis, a multivariate model was constructed to assess the influence of patient covariates on continuous outcomes: length of ICU stay (Poisson regression), number of units transfused (ordinal logistic regression) and total hospital stay (Poisson regression). MIS was found to be a predictor of reduced stay in the ICU (coef − 3.25, 95% CI [− 4.93, − 0.59], *p* = 0.036) as was the pre-operative Euroscore (coef 0.16, 95% CI [0.05, 0.29], *p* < 0.001) (Table 4A). Cross clamp time, CPB time and patient age were all found to have no influence on the length of ICU stay.

**Table 4:** (A) Multivariate regression analysis: ICU stay (days) (Poisson regression analysis), (B) Multivariate regression analysis: Blood Units transfused (results of ordinal logistic regression analysis), (C) Multivariate regression analysis: Total hospital stay (days) (Poisson regression analysis)

	Coef	Standard error	95% CI	*p* value
(A)				
Mini-sternotomy	− 3.25	1.30	− 4.93 to − 0.59	**0.036**
Euroscore	0.16	0.10	0.05 to 0.29	**< 0.001**
AXT	0.021	0.002	− 0.006 to 0.051	0.280
CPB	0.006	0.001	− 0.012 to 0.015	0.354
Age	0.024	0.054	− 0.032 to 0.053	0.631
(B)				
Mini-sternotomy	− 0.10	0.64	− 1.35 to 1.16	0.877
Euroscore	− 0.26	0.47	− 1.18 to 0.65	0.575
CPB time	0.053	0.032	− 0.0095 to 0.12	0.097
Cross clamp time	− 0.058	0.040	− 0.14 to 0.019	0.141
(C)				
Mini-sternotomy	− 2.70	0.92	− 4.52 to − 0.87	**0.004**
LV function	− 0.13	0.05	− 0.23 to − 0.03	**0.013**
COPD	1.38	1.53	− 1.677 to 4.44	0.371
Diabetes	− 0.83	1.10	− 3.03 to 1.37	0.454
Renal failure	5.06	2.11	− 0.85 to 9.27	0.190

Bold type indicates statistical signifiance

Comparatively, none of the tested covariates were found to be significant predictors of the number of units of blood transfused (mini-sternotomy, Euroscore, CPB times and cross clamp times) (*p* > 0.05) (Table 4B).

MIS was also a strong predictor of reduced total hospital stay (coef − 2.70, 95% CI [− 4.52, − 0.87], *p* = 0.004), as was favourable pre-operative LV function (coef − 0.13, 95% CI [− 0.23, − 0.03], *p* = 0.013) (Table 4C). Other co-morbidities were found to have no influence on hospital stay.

### Echocardiographic follow-up

Early follow-up echocardiography found a significant improvement in effective orifice area, left ventricular dimension and volume indices, and LVEF (*p* > 0.05) for all patients. Multivariate analysis was conducted to compare relevant pre-operative covariates (BMI, BSA and Euroscore) as well operative covariates (prosthesis size and surgical approach) with important echocardiographic outcomes. Importantly, the analysis found mini-sternotomy to be a predictor for reduced LV diastolic volume (coef − 0.35, 95% CI [− 1.02, − 0.05], *p* = 0.05) by 6 months follow-up (Table 5).

**Table 5:** Multivariate regression analysis: predictors of echocardiographic outcomes at 6–12 months follow up

	Coef	Standard error	95% CI	*p* value
*LVEF*				
Mini-sternotomy	5.22	2.92	− 0.70 to 11.16	0.182
Euroscore	− 0.50	0.47	− 1.44 to 0.45	0.297
Prosthesis size	− 1.74	1.49	− 3.76 to − 1.29	**0.043**
Age	0.30	0.28	− 0.26 to 0.86	0.279
*EOA*				
Mini access	− 0.04	0.10	− 0.25 to 0.16	0.670
Euroscore	− 0.03	0.01	− 0.05 to − 0.04	**0.050**
Prosthesis size	− 0.02	0.05	− 0.11 to 0.10	0.891
*End diastolic volume*				
Mini-sternotomy	− 0.35	0.34	− 1.02 to − 0.05	**0.050**
Euroscore	− 0.37	0.18	− 0.73 to 0.04	0.070
Prosthesis size	− 0.04	0.13	− 0.33 to 0.24	0.759
BMI	− 0.04	0.04	− 0.12 to 0.05	0.377
BSA	0.30	0.85	− 1.51 to 2.11	0.729
*End systolic volume*				
Mini-sternotomy	0.37	0.41	− 0.49 to 1.23	0.384
Euroscore	− 0.42	0.23	− 0.90 to 0.06	0.081
Prothesis size	0.02	0.18	− 0.36 to 0.39	0.922
BMI	0.01	0.06	− 0.12 to 0.13	0.938
BSA	0.18	1.22	− 2.41 to 2.76	0.886

Bold type indicates statistical signifiance

Two other positive findings were: the negative effect of prosthesis size on LV function at follow-up (coef − 1.74, 95% CI [− 3.76, − 1.29], *p* = 0.043) and. The improved effect of a lower Euroscore on the effective orifice area (coef − 0.04, 95% CI [− 0.15, − 0.02], *p* = 0.05). Patient BMI and BSA were were found not to influence post-oeprative echocardiographic outcomes.

## Discussion

Potentiating minimal access surgery for treating aortic valve disease is likely to develop in the coming years with sutureless valves playing an important role. The drive to minimise sternal trauma is furthered by the enhancement of transcatheter aortic valve implantation (TAVI). The recent expansion of risk-categories encompassed by TAVI treatment [6] continues to provide a significant alternative to all forms of surgical AVR, by avoiding sternal trauma altogether. However, the challenges associated with TAVI (poor femoral vessel access, multi-valve surgery and severe mitral annular calcification) as well as higher complications of paravaulvular leak and heart block, necessitates constant development in surgical approaches, and indeed, the above challenges can at times contraindicate patients for TAVI treatment altogether.

Only a few studies provide comparative data between the outcomes of sutureless valves in combination with mini-sternotomy versus conventional access [2, 8, 9]. The additive effect of sutureless technology to mini-sternotomy access can potentially circumvent the technical challenge of reduced operative space, hence limiting the increased operative time usually associated with mini-AVR. Our study has found equivocal operating times between conventional and mini-sternotomy, which contrasts with many studies that found longer cross clamp times in mini-AVR with sutured valves. Furthermore, the rates of short-term complications were similar between both cohorts, which is also echoed by our results.

### Benefits of sutureless valves

Rapid deployment valve technology has been a primary innovation in surgical AVR in the last two decades based on the design of transcatheter valves [10]. The self-expandable, stentless and sutureless Perceval S (Sorin Group Italia Srl, Saluggia, Italy) and the balloon-expandable, stented Intuity valve (Edwards Lifesciences, Irvine, CA) are the most frequently implanted sutureless valves worldwide for AVR.

The technology exhibited by sutureless valves afford it two main recognised benefits: (1) ergonomic implantation (particular effective in minimally invasive surgery) and (2) favourable valve hemodynamics (particularly with Perceval) [3, 11]. The valve design eliminates the need for sutures to be placed in the aortic annulus (aside from 3 guiding stitches), which can typically take 15–20 min. This added benefit reduces cardiopulmonary bypass time and its associated complications, especially in high-risk patient groups.

Numerous studies have compared the outcomes of sutureless valves with conventionally implanted AVRs. Postoperative bleeding and blood transfusion requirements have also been reported to diminish, which likely reflects the shorter time on the CPB machine [12–14]. There has also been reported reductions in ventilation time [12, 13], reduced incidence of acute kidney injury [13, 14], shorter intensive care stay [12] and shorter hospital stay [12].

Many studies have reported a higher rate of permanent pacemaker (PPM) insertion after Perceval valve implantation [13–16]. A recent meta-analysis by Sohn and colleagues (21 studies, n = 2785) reaffirmed the higher rate of PPM insertion in sutureless valves compared to conventional prostheses (relative risk 2.08; 95% CI, 1.49–2.90) [17].

When compared to TAVI, Santarpino and colleagues [18] conducted a propensity matched analysis (n = TAVI 538 vs sutureless 385) and showed that sutureless valves resulted in better long-term outcomes compared to TAVI, despite the increased need for blood transfusions in the short term. Furthermore, one randomised trial of TAVI with an early-generation valve in 280 patients demonstrated that TAVI was not inferior to surgery with more than 5 years of follow-up [19]. In addition to this, a meta-analysis found that sutureless valves result in improved perioperative survival compared to TAVI, albeit with only 6 studies analysed, adding further weight to case of sutureless valves as a viable option, especially for minimally invasive approaches [20].

### Enhanced recovery with sutureless via minimal access

In the present study, mini-sternotomy was found to be a strong predictor of shorter ICU stay (*p* = 0.024), which concords with similar large studies. A recent multi-centre study based on propensity matched data from the STS registry [21] compared 1,341 AVR patients in two cohorts: conventional sternotomy vs mini-AVR (either through partial sternotomy or right mini-thoracotomy). Mini-AVR demonstrated enhanced recovery through decreased ventilator time (5 vs 6 h; *p* = 0.04) and earlier discharge (15.2% vs 4.8% in ≤ 4 days; *p* < 0.001). The study also found a lower rate of blood transfusion which was not seen in our patients. Interestingly, we found the rate of new onset atrial fibrillation (AF) post-surgery in mini-AVR to be almost half of that in the sternotomy cohort (24% vs 42%) although this did not reach significance (*p* = 0.250).

On the other hand, studies reporting non-superiority of MIS compared to sternotomy with regards to enhanced recovery have also been published. A recent RCT led by Papworth (UK), (Mini-Stern trial) (N = 118 MS vs N = 118 MIS, all conventional sutured prostheses in both cohorts) [22] found no added benefits offered by mini-sternotomy for recovery time, rather minimal access resulted in longer CPB and cross clamp times. This perhaps gives precedent for the use of operative adjuncts, such as sutureless valves, to augment the benefit of minimal access surgery.

### Early myocardial remodelling

The present study has also demonstrated a significant positive impact of mini-AVR on left ventricular dimensions by 6 months. This could reflect the reduced myocardial handling intra-operatively and improved patient pain profile, leading early cardiovascular recovery and rehabilitation, allowing the myocardium to adapt to the haemodynamic changes affected by the treated aortic valve [23]. This contrasts with other reports in the literature [3]: the study by Dalen and colleagues, compared mini-sternotomy patients with median sternotomy (all receiving isolated Perceval AVR—like the present study), and the most notable finding was a significantly higher post-operative transvalvular gradient (28.1 vs 23.3 mmHg, *p* = 0.026) in the mini-AVR group.

### Limitations

The main drawback of the present study is the small sample size, which limits the power of our conclusions (e.g. shorter ICU/hospital stay). Whilst the outcomes for our unit was very positive for major short-term complication (zero incidence of mortality and stroke) this may also reflect the underpowered cohorts, which limits their ability to detect potential differences. This is particularly the case for post-operative AF, where the lower incidence in the mini-AVR group, although non-significant, may provide clinical relevance in a larger patient group. Although our follow-up time is limited to one year, the echocardiographic findings we have provided are unique and warrant further exploration in a larger patient group over a longer period. Indeed, long-term echocardiographic results for the Perceval valve, whilst mostly promising, has raised questions over the durability of its nitinol frame after a few years. This could also be correlated with patient functional outcome, which the present study did not analyse.

## Conclusions

The study provides an insight to the benefit of combining two important technologies in AVR: mini-sternotomy, and sutureless valves. The equivocal operative times, enhanced recovery and improved early myocardial remodelling provides potentially improved outcomes for treating aortic stenosis compared to mini-AVR or sutureless valves in isolation.

## Data Availability

Not applicable.
